# Complete mitochondrial genome of *Agapornis lilianae* (Psittaciformes: Psittacidae), with its phylogenetic analysis

**DOI:** 10.1080/23802359.2019.1675552

**Published:** 2019-10-12

**Authors:** Yun-Xia Chen, Yong-Wu Zhou, Sen-Lin Hou, Ya-Lin Huang

**Affiliations:** a Nanjing Forest Police College, Nanjing, China;; b Key Laboratory of Wildlife Evidence Technology State Forest and Grassland Administration, Nanjing, China

**Keywords:** Lilian’s Lovebird, *Agapornis lilianae*, mitochondrial genome, phylogenetic analysis

## Abstract

In this paper, we reported the complete mitogenome of *Agapornis lilianae*. *A. lilianae* mitogenome is 16,720bp base pairs long. The overall base compositions of complete mitogenomes of *A. lilianae* is 29.66% A, 21.5% T, 34.59% C, 14.25% G. All genes exhibit the typical mitochondrial gene arrangement and transcribing directions. The phylogenetic analysis based on 21 mitochondrial sequence data suggested that *A. lilianae* are closely related to the same genus specie *Agapornis roseicollis*. The results obtained here would be useful for the conservation and phylogeny of *A. lilianae*.

*Agapornis lilianae*, also known as Lilian’s Lovebird, belonging to the family Psittacidae (Aves, Psittaciformes). This specie is mainly distributed in Malawi, Mozambique, United Republic of Tanzania, Zambia and Zimbabwe (IUCN 2018), mainly inhabits valleys filled with trees and prefers *Acacia* plants. Sometimes they will be active in the forest areas between 600 and 1000 metres, or go to highland forest areas, plain areas and grassland according to different seasons (Mzumara et al. 2014; IUCN 2018). The bright and colourful feathers, and easy to breed, lots of *A. lilianae* were caught and bred in captivity as pet. In recent years, the wild population of *A. lilianae* has declined (Soobramoney and Perrin 2014; Mzumara et al. 2016; IUCN 2018).

Whole blood sample was collected from one individual of *A. nigrigenis*, which was bred in the Nanjing Hongshan Forest Zoo (N32°09′, E118°80′), Jiangsu province, China. Genomic DNA was isolated using the DNAiso reagent (Takara, Beijing, China) and was stored in the Forest Police Forensic Centre of State Forest Administration (Accession S2019J1101203). A set of universal primers were designed for polymerase chain reaction amplification, and Sanger sequencing was performed based on the complete mitogenomes of *Agapornis roseicollis* (GenBank accession: EU410486.1).

The complete mitochondrial genome (GenBank accession: MN481406) of *A. lilianae* is a typical circular DNA molecule with 16,720bp in length. The nucleotide composition of the genome is 29.66% A, 21.5% T, 34.59% C, 14.25% G, with A + T content of 51.16%. Mitochondrial genome of *A. lilianae* was composed with 13 protein-coding genes, 22 transfer RNA genes, 2 ribosomal RNA genes, and a non-coding control region. The gene arrangement and transcribing directions in the mitochondrial genome of *A. lilianae* is identical to other lovebirds (Eberhard and Wright 2016; Liu et al. 2019).

Phylogenetic analysis of *A. lilianae* was performed based on the complete mitogenome of 21 parrot species. Sequence dataset were aligned using ClustalX and Neighbour-Joining (NJ) analysis was conducted using MEGA 7.0, with 1000 bootstrap replicates (Kumar et al. 2016). The phylogenetic NJ tree showed that the mitogenome of *A. lilianae* was genetically the closest to the *Agapornis roseicollis* of the same genus (Figure 1). The results obtained here could contribute to future studies on population genetics and biological conservation of Lovebirds.

**Figure 1.**
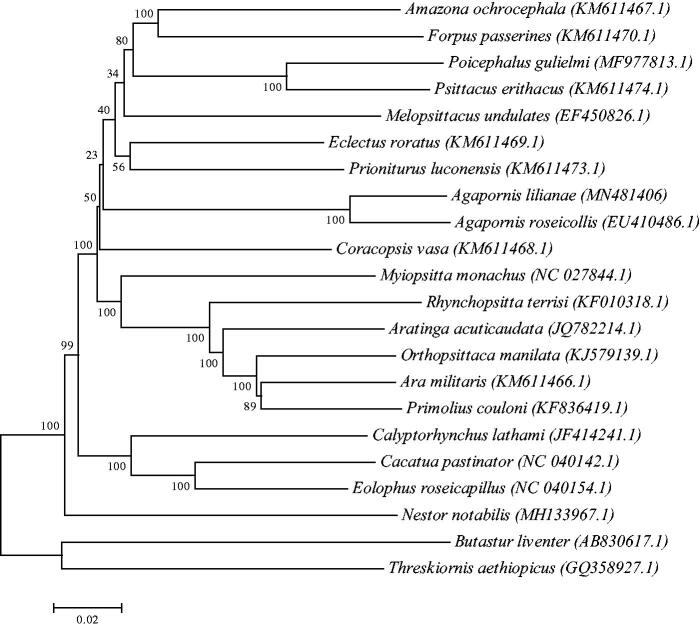
Neighbour-joining phylogenetic tree based on the complete mitogenomes of 21 parrot species, constructed using MEGA 7.0. Numbers following scientific names are GenBank accessions.
